# Transformation improvement with the Standardized Pressure A*grobacterium* Infiltration Device (SPAID)

**DOI:** 10.1186/s13104-019-4117-3

**Published:** 2019-03-15

**Authors:** Mohamad Fadhli bin Mad’ Atari, Kevin M. Folta

**Affiliations:** 1 Horticultural Sciences Department, University of Florida, 1301 Fifield Hall, PO Box 110690, Gainesville, FL 32611 USA; 2 Present Address: School of Biological Sciences, Universiti Sains Malaysia, 11800 Penang, Malaysia

**Keywords:** Transient, Stable, Transformation, Agrobacterium, Diploid strawberry, Adventitious shoots, *Fragaria vesca*, *Nicotiana benthamiana*, *Arabidopsis thaliana*

## Abstract

**Objective:**

The treatment of plant tissue with *Agrobacterium tumefaciens* is often a critical first step to both stable and transient plant transformation. In both applications bacterial suspensions are oftentimes physically introduced into plant tissues using hand-driven pressure from a needleless syringe. While effective, this approach has several drawbacks that limit reproducibility. Pressure must be provided with the syringe perfectly perpendicular to the tissue surface. The researcher must also attempt to provide even and consistent pressure, both within and between experimental replicates. These factors mean that the procedures do not always translate well between research groups or biological replicates.

**Results:**

We have devised a method to introduce *Agrobacterium* suspensions into plant leaves with greater reproducibility. Using a decommissioned dissecting microscope as an armature, a syringe body with the bacterial suspension is mounted to the nosepiece. Gentle, even pressure is applied by rotating the focus knob. The treatment force is measured using a basic kitchen scale. The development of the Standardized Pressure Agrobacterium Infiltration Device (SPAID) provides a means to deliver consistent amounts of bacterial suspensions into plant tissues with the goal of increasing reproducibility between replicates and laboratories.

## Introduction

The ability to introduce *Agrobacterium tumefaciens* cells into plant tissues the cornerstone of many transient [1–5] and stable [6] transformation procedures. *Agrobacterium* executes a genetic exchange with the plant cell it infects, enabling the plant cell, and by extension any tissues arising from it, to express the gene(s) introduced. *Agrobacterium*-mediated transformation protocols require incubation of plant tissues with bacterial suspensions. The process has been optimized using several methods, including floral dipping [7–9], vacuum infiltration [10], sonication [11, 12] air brushes [13], or introduction of bacteria by forcing suspensions into leaves under pressure.

Transient expression in plants like *N. benthamiana* is a useful way to test the activity of promoters [14], localization [15], or interacting partners [16] for a given protein. This system also allows production of industrial proteins [17, 18] or the effect of a specific introduced gene on a transcriptome [19]. *Agrobacterium* suspensions are typically introduced using a needleless syringe, the aperture pressed against the abaxial leaf surface and then pressure applied by hand [20]. Successful introduction leads to a media-soaked lesion. The bacteria perform their genetic exchange and express the protein of interest. While crude, the method is effective.

However, as transient assays attempt to resolve more sophisticated questions, the crude ‘push-n-pray’ methods could benefit from standardization. The current protocols have several limitations. A failure to make perfect perpendicular contact between the syringe tip and the leaf surface leads to variation between infiltration events. Too much pressure also can cause physical damage to the leaf surface that can affect results and permit introduction of other pathogens. Whether the end goal is transient expression or stable transformation, a method providing consistent introduction of bacterial suspensions may be helpful.

**Fig. 1:**
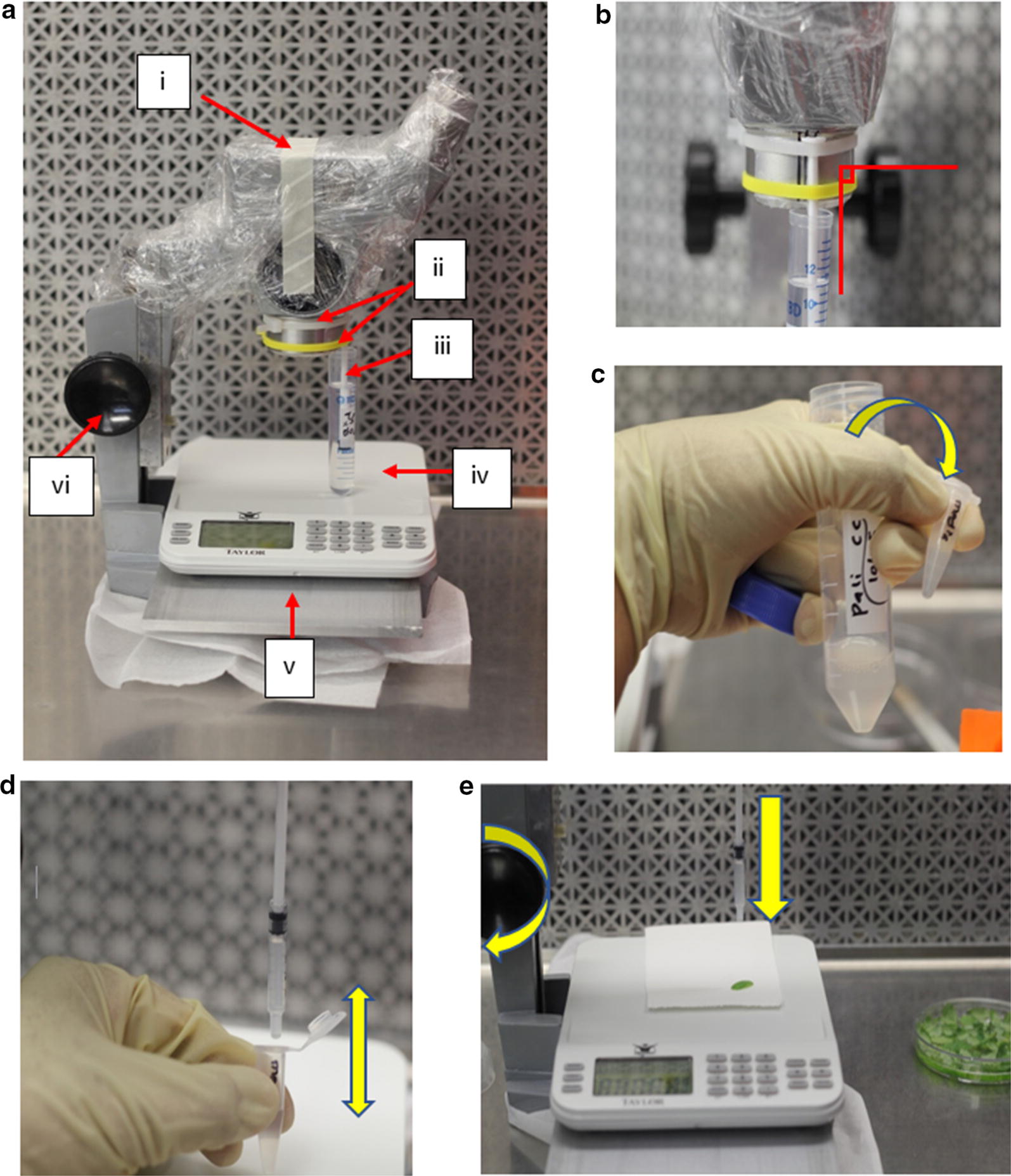
Attributes of the Standardized Pressure Agroinfiltration Device (SPAID). **a** The following labeled parts: (i) Plastic wrap, (ii) Cable ties holding syringe plunger, (iii) Syringe plunger soaking in 95% ethanol, (iv) Electronic analytical balance (in grams), (v) Metal plate, (vi) Dissecting microscope with coarse adjustment knob. **b** The syringe plunger mounted to the microscope nosepiece perpendicular to the microscope stage. **c** *Agrobacterium* solutions are transferred to a microcentrifuge tube. **d** *Agrobacterium* solutions are drawn into a syringe body. **e** Leaf explants are treated with *Agrobacterium* solution by rotating the coarse adjustment knob while monitoring reading on electronic scale

In this report we describe the Standardized Pressure Agrobacterium Infiltration Device (SPAID). The mechanism is a simple modification of a defunct dissecting microscope used in tandem with an analytical balance. The microscope nosepiece is fit with a permanent syringe plunger (Fig. 1), and bacterial suspensions are loaded into a sterile syringe body. Rotation of the focus knob allows for precise contact and pressure with the leaf surface. This instrument provides a means to standardize pressure and leaf contact angle, significantly reducing variation in the delivery of *Agrobacterium* solutions into plant tissues. Consistent introduction of bacterial solutions may lead to optimized expression of transient constructs or improved stable transformation rates.

## Main text

### Design and application of the SPAID

Precise, linear control of pressure on the subject was obtained by mounting a syringe plunger to the nosepiece of a decommissioned dissecting microscope (Fig. 1a). The dissecting microscope objectives were removed and a syringe plunger was attached to the nosepiece using cable ties (Fig. 1b). The microscope frame was cleaned with 70% ethanol, and all subsequent procedures were performed in a laminar flow hood. The plunger was oriented in a perfectly vertical position and remained attached to the microscope between trials. It was replaced only after many uses when it failed to create a complete seal within the syringe body.

Syringe bodies were cut to approximately half their original size and stored in 95% ethanol. For each treatment, a syringe body was dried by evaporation in a laminar flow hood and mounted onto the plunger (Fig. 1d). The Agrobacterium suspension is drawn into the syringe by pulling down on the syringe body with sterile forceps while the tip is submerged in culture.

Sterile leaf explants were then treated by rotating the focus knob and touching the tip of the syringe on to the leaf surface. The pressure applied was measured using an analytical balance (Fig. 1e).

### Plant materials

All plant materials are common laboratory genotypes used in plant transformation. Strawberry (*Fragaria vesca*, ‘YW5AF7’) and tobacco (*Nicotiana benthamiana*) leaf explants were obtained from materials grown in magenta culture containers in ½ × Murashige and Skoog media (pH 5.8) solidified with 0.8% phytoagar (Phytotechnology Laboratories, Shawnee Mission, KS). Individual leaves were excised under a laminar flow hood with sterile forceps, and floated on sterile water. *Arabidopsis thaliana* leaves were obtained from plants (ecotype Col-0) grown aseptically on the same media in deep petri dishes.

### Agrobacterium culture conditions

All *Agrobacterium* cultures (strain GV3101) containing the plasmid pKWGD7, containing a constitutive promoter fused to green fluorescent protein (GFP), were grown at 29 °C in Luria Broth containing 5 g NaCl/L to 0.8 OD_600_. The cultures were centrifuged at 3000×*g* for 10 min and resuspended in ½ × Murashige and Skoog (MS) medium pH 5.6 to a specified density prior to SPAID-mediated inoculation.

### Post-infiltration treatment

Explants were moved to solid medium containing plant growth regulators to induce callus formation. Strawberry explants were moved to the media defined in Slovin et al. [21]. Tobacco explants were cultured on “selection medium” [22] and Arabidopsis explants were cultured on Callus Induction Medium [23]. For tobacco and Arabidopsis the media prepared substituted phytoagar (RPI, Mt. Prospect, IL) for solidification. GFP positive calli and foci were counted after 2 weeks.

### Tests of culture density

*Agrobacterium* cultures were applied to strawberry and tobacco leaf explants at different concentrations to test for effect of culture density on transformation at a medium delivery pressure. *Agrobacterium* cultures were resuspended in 1/2 × MS to obtain final culture densities of 0.1, 0.5, 0.8, or 1.2 OD_600_. Each suspension was applied to abaxial leaf surfaces at 273 kilopascals (kPa; a reading of 500 g on the balance) for 5 s. The results from strawberry showed no significant difference between culture densities (Table 1). When used on tobacco, 100% of explants featured GFP positive calli and many foci, and results were not significant between treatments (not shown).

**Table 1:** *Agrobacterium* concentration effects on transformation of strawberry leaf explants

OD_600_	% of explants with GFP callus	% of explants with GFP foci
0.1	18.9 ± 4.8 a	49 ± 9.5 a
0.5	21.1 ± 5.4 a	43.3 ± 6.0 a
0.8	21.1 ± 4.5 a	46.7 ± 8.2 a
1.2	18.9 ± 4.6 a	42.2 ± 5.8 a

Thirty leaf explants for each concentration were used, and the experiment was repeated three times. The percent of surface area was calculated by using Quant Software. Data analyses were conducted with ANOVA by proc glm procedure SAS 9.4 with α = 0.05. The total surface area calculated is 0.401 cm^2^

### Tests of pressure on transformation rate

Agrobacterium cultures (0.5 OD_600_) were then applied to the abaxial side of leaf explants using various pressures. The pressure applied at the syringe tip was calculated in kPa based on the area of the syringe opening using values obtained from an analytical balance. Forces (kPa) applied to the explants were 54 (100 g), 109 (200 g), 163 (300 g), 218 (400 g), 273 (500 g), 327 (600 g), and 546 (1000 g). Stable transformation was scored as the number of GFP foci 2 weeks after treatment. The results are presented in Table 2. For strawberry, there were significant differences through the mid-range of pressures tested (500–800 kPa). For tobacco, lower and mid-range pressures (< 600 kPa) provided the greatest numbers of GFP-positive calli, and Arabidopsis also showed more explants with stable foci when treated with lower pressures (< 600 kPa). Visible damage to leaves was observed frequently in tobacco and Arabidopsis above 800 kPa (not shown).

**Table 2:** Relationship between application pressure and stable GFP-positive foci on leaf explants from four plant species

Scale reading (g)	kPa	Percent of explants with stable GFP foci		
		Strawberry	Tobacco	Arabidopsis
100	54	8.89 ± 2.10 a	84.50 ± 8.96 a	35.55 ± 4.33 a
200	109	9.68 ± 2.10 a	80.49 ± 6.95 a	22.06 ± 3.59 a
300	163	17.64 ± 4.10 ab	77.66 ± 6.18 a	24.49 ± 7.62 a
400	218	17.97 ± 11.1 ab	77.97 ± 7.34 a	21.92 ± 1.42 a
500	273	49.04 ± 11.1 c	85.04 ± 12.91 ab	16.09 ± 5.05 ab
600	327	40.49 ± 5.65 c	70.49 ± 3.68 a	9.86 ± 9.80 ab
700	382	31.33 ± 5.14 b	40.33 ± 5.92 b	8.23 ± 3.83 b
800	437	37.67 ± 10.36 bc	45.67 ± 9.90 b	11.35 ± 1.63 b
900	491	7.49 ± 1.95 a	27.49 ± 2.41 c	8.46 ± 3.47 b
1000	546	11.00 ± 4.35 a	21.00 ± 6.38 c	9.66 ± 2.23 b

GFP positive callus events were counted 2 weeks after cocultivation with different syringe pressures applied on strawberry, tobacco or Arabidopsis leaf explants. Three separate experimental replicates were performed containing 30–40 explants each. Data analyses were conducted with ANOVA by proc glm procedure SAS 9.4 with α = 0.05

### Conclusions

In the post-genomics era methods are required to test candidate gene function, localization and protein–protein interaction partners in vivo. While stable transformation is the gold standard, transient expression methods can be relatively rapid, high throughput, yet meaningful. However, these methods are limited in part by the inconsistency between experimental replicates. This may be attributed to the relatively simple means used to introduce the transforming bacteria into plant tissues—simply pressing in culture through a needleless syringe. In our hands, these methods suffer from extreme variability and low reproducibility, and while results are suggestive, there is a need to limit the sources of variation in the assay. The goal of the current work was to control as many variables as possible in the agroinfiltration process, with the goal of decreasing variability inherent in transient assays, as well as optimizing transformation frequencies.

Previous work has examined the roles of culture density, *Agrobacterium* strain, and various additives (such as ascorbate or acetosyringone) [24]. Many other reports detail introduction of cultures based on a variety of methods from vacuum infiltration to sonication. In all of these delivery methods parameters may be controlled precisely, allowing some control of culture into the subject tissue. However, hand delivery of bacterial suspensions into tissue via a needleless syringe is not as easily controlled for consistency.

The SPAID removes a central issue in syringe-mediated agroinfiltration—the perpendicular introduction of bacterial suspensions to the leaf surface. When performed by hand, imperfect seals or uneven pressure on the leaf surface causes liquid to leak from the leaf-syringe interface, leading to less introduction into the leaf variation in pressure between trials. Moreover, when higher pressures are applied, the edges of the syringe opening can damage the leaf, leading to a necrotic lesion that could affect explant vitality and ultimately transformation and regeneration rates.

Several variables were tested in strawberry, tobacco and Arabidopsis. Different concentrations of *Agrobacterium* suspension were tested, based upon values that are commonly used in plant transformation. The results show no difference between relatively common levels used, suggesting that culture density is not an important variable and even lower concentrations may be successfully utilized in these experiments.

On the other hand, differences in stable transformation were observed, and were pressure dependent. Strawberry responded best to pressures in the middle of the range tested. Tobacco and Arabidopsis responded best to lower pressures. These findings are important and underscore the utility of the SPAID approach, as less pressure is may be better for balancing bacterial introduction and tissue injury, yet is extremely difficult to accurately achieve with manual application. This approach may be extremely valuable when *Agrobacterium* cultures are introduced to non-detached leaves, as is the case with many applications in *N. benthamiana*.

The use of the SPAID approach may be especially useful when RNAseq is being performed on transiently transformed tissues. The report by Bond et al. [19] examines gene expression in leaf cells of *M. truncatula* and *N. benthamiana* when infiltrated with *Agrobacterium* cultures containing a specific MYB-type transcription factor. The results showed expected changes in target genes, but also unveiled discovery of new regulators and feedback mechanisms. Such approaches are clearly effective, and will only improve with standardization through use of consistently applied treatments, such as those enabled with the SPAID.

The work presented here takes on the first step—introducing the transgene at a high rate. This process removes one source of variability and error that ultimately may affect rate-limiting downstream processes, such as regeneration of plantlets. Additional applications in transient gene expression, protein–protein interaction, and protein localization also may benefit from use of this delivery device.

## Limitations

As demonstrated in “Main text” section, the optimization must be performed on each type of plant and each type of tissue studied. The device also requires use of a sterile environment such as a laminar flow hood, which may not be accessible to some users.

## Abbreviations

SPAID: Standardized Pressure Agrobacterium Infiltration Device
GFP: green fluorescent protein
MS: Murashige and Skoog (medium)
kPa: Kilopascals, SI unit of pressure
